# Temperature evolution of the high-harmonic magnetic modulations in DyFeO_3_

**DOI:** 10.1107/S160057672600508X

**Published:** 2026-07-22

**Authors:** Piotr Fabrykiewicz, Dnyaneshwar R. Bhosale, Martin Meven, Astrid Schneidewind, Karin Schmalzl, Michał Stękiel

**Affiliations:** a Jülich Centre for Neutron Science at Heinz Maier-Leibnitz Zentrum, Forschungszentrum Jülich GmbH, Lichtenbergstraße 1, 85747 Garching, Germany; b Institute of Crystallography, RWTH Aachen University, Jägerstraße 17-19, 52066 Aachen, Germany; c Jülich Centre for Neutron Science at Institut Laue–Langevin Forschungszentrum Jülich GmbH 71 Avenue des Martyrs 38000 Grenoble France; Technical University of Denmark, Denmark

**Keywords:** DyFeO_3_ orthoferrite, incommensurate magnetic order, high-order satellite reflections, soliton lattice, neutron diffraction

## Abstract

We report on the temperature evolution of magnetic satellites associated with the incommensurate magnetic ordering of Dy^3+^ ions in DyFeO_3_ below 4.1 K. Using high-resolution neutron diffraction, incommensurate magnetic reflections, satellites of the 001 reflection with odd harmonics up to the seventh order, were observed and their temperature dependence quantified. Key parameters, such as the modulation length, FWHM and integrated intensities, were analysed across the measured temperature range. The results were compared with a previous study of the soliton lattice in TbFeO_3_ which appears under an applied magnetic field. Possible microscopic models underlying the magnetic order of Dy^3+^ are discussed in this context.

## Abstract 2

The temperature evolution of the high-harmonic incommensurate magnetic ordering of Dy^3+^ in DyFeO_3_ orthoferrite is compared with the literature results on the soliton lattice of TbFeO_3_ observed in an external magnetic field.

## 1. Introduction

DyFeO_3_ is the only known rare earth orthoferrite with an incommensurate ground state (Mareschal & Sivardière, 1969 ▸; Ritter *et al.*, 2022 ▸; Biswas *et al.*, 2022 ▸). Below the Dy Néel temperature 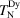 ≃ 4 K, it exhibits multiferroic properties such as the giant magnetoelectric effect (Tokunaga *et al.*, 2008 ▸; Wang *et al.*, 2016 ▸) and the piezomagnetoelectric effect (Nakajima *et al.*, 2015 ▸). It was also shown that spin dynamics in DyFeO_3_ can be controlled via the inverse Faraday effect (Kimel *et al.*, 2005 ▸). Understanding the connection between these properties and the incommensurate ground state requires a thorough characterization of the magnetic order of DyFeO_3_ at low temperatures.

DyFeO_3_ crystallizes in an orthorhombically distorted perovskite structure, which is described by the *Pbnm* space group (non-standard setting of *Pnma*, No. 62) (Eibschütz, 1965 ▸; Marezio *et al.*, 1970 ▸). Below the Fe^3+^ Néel temperature of 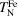 ≃ 645 K the magnetic moments of the Fe^3+^ ions establish a 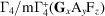 structure [first and second irreducible representation (irrep) labels are according to Bertaut (1963 ▸) and Cracknell, Davies, Miller & Love (CDML) (Miller & Love, 1967 ▸; Cracknell *et al.*, 1979 ▸) notations, respectively]. This has magnetic space group (MSG) *Pb*′*n*′*m*, *i.e.* a nonstandard setting of MSG *Pn*′*ma*′, BNS No. 62.448. Below the spin reorientation temperature [30–80 K, exact values vary depending on the study; Ritter *et al.* (2022 ▸) and citations within], the Fe^3+^ ions establish a 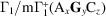 magnetic order (MSG *Pbnm*.1, *i.e.* a nonstandard setting of MSG *Pnma*.1, BNS No. 62.441). The reorientation of the **G** component and the disappearance of the F component have both been confirmed by neutron diffraction (Mareschal & Sivardière, 1969 ▸; Wang *et al.*, 2016 ▸; Ritter *et al.*, 2022 ▸) and magnetization studies (Bozorth *et al.*, 1958 ▸; Zhao *et al.*, 2014 ▸; Wang *et al.*, 2016 ▸; Biswas *et al.*, 2022 ▸).

Early studies (Nowik & Williams, 1966 ▸; Gorodetsky *et al.*, 1967 ▸; Gorodetsky *et al.*, 1968 ▸; Belov *et al.*, 1968 ▸; Berton & Sharon, 1968 ▸; Schuchert *et al.*, 1968 ▸) described the Dy^3+^ magnetic structure in DyFeO_3_ below 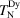
 ≃ 4 K as a com­men­surate 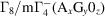 ordering. A neutron powder diffraction study by Mareschal & Sivardière (1969 ▸) showed that the Dy^3+^ ordering is incommensurate, as evidenced by a slight splitting of the 001 reflection. However, some recent reports still use the commensurate description, but in the form of a 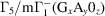 representation (Tokunaga *et al.*, 2008 ▸; Rajeswaran *et al.*, 2013 ▸; Zhao *et al.*, 2014 ▸; Nakajima *et al.*, 2015 ▸; Wang *et al.*, 2016 ▸; Stanislavchuk *et al.*, 2016 ▸; Hoogeboom *et al.*, 2021 ▸). The Dy^3+^ incommensurate ordering in DyFeO_3_ was not discussed for half a century but has been recently addressed by Ritter *et al.* (2022 ▸) and Biswas *et al.* (2022 ▸) with neutron powder diffraction. The authors of those reports proposed three different models of the Dy^3+^ magnetic ordering with modulation vector (0, 0, δ): (i) a transverse spin-density wave [magnetic superspace group (MSSG) *Pbnm*.1(0, 0, γ)*s*00, 62.1.9.4.m441.1; Ritter *et al.*, 2022 ▸], (ii) an elliptical-based helical ordering [MSSG *Pbn*2_1_.1(0, 0, γ)*s*0*s*, 33.1.9.2.m144.1; Ritter *et al.*, 2022 ▸] and (iii) a spin-density wave coexisting with commensurate 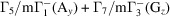 ordering [MSSG *Pn*′(α0γ)0, a nonstandard setting of MSSG *Pb*′(α, β, 0)0, 7.1.2.1.*m*26.1; Biswas *et al.*, 2022 ▸]. Model (iii) is highly improbable, as it results in Dy^3+^ magnetic moments ranging from 6.6 to 14.2 µ_B_, with the latter value exceeding the expected magnetic moment of the isolated Dy^3+^ ion, *i.e.* 10 µ_B_. In the helical model (ii) (Ritter *et al.*, 2022 ▸), half of the Dy magnetic moments rotate clockwise and the other half rotate anticlockwise. This results in a change in the angle between Dy magnetic moments in neighbouring helices from one unit cell to the next. This seems rather unlikely and was not discussed further by Ritter *et al.* (2022 ▸). In fact, it was shown (Fabrykiewicz *et al.*, 2021 ▸) that helical ordering with one global chirality is only possible in the Sohncke magnetic groups, *i.e.* space groups without inversion centres, mirrors or glide planes. This makes the chiral magnetic ordering incompatible with the *Pbnm* space group of DyFeO_3_. In addition, the Dy^3+^ ions show a very strong Ising-like anisotropy (Zvezdin & Matveev, 1979 ▸), which supports the validity of the spin-density wave model (i). However, all these models introduce a modulation of the magnetic moment amplitude of Dy. This would be typically caused by the Kondo effect, a screening of the 4*f* orbital by conduction-band electrons, but DyFeO_3_ is insulating and does not allow for such an effect.

Therefore, high-resolution single-crystal neutron diffraction on DyFeO_3_, as already mentioned by Mareschal & Sivardière (1969 ▸) and recently readdressed by Ritter *et al.* (2022 ▸), is highly needed to characterize the Dy^3+^ incommensurate ordering in detail. A recent study by Nikitin *et al.* (2025 ▸) discusses this issue and, based on the point-charge model of the crystal electric fields, suggests collinear ordering of Dy moments. Further, it presents a numerical model of the domain structure in DyFeO_3_ that explains the multiple satellites of the 001 reflection and follows its evolution in the applied field. Our results are in line with those of Nikitin *et al.* (2025 ▸). In this study, we focus on the temperature evolution of the domain distribution based on the characteristics of the satellite reflections, that is, their intensities and widths. We provide analysis of the key parameters as a function of temperature and interpret them in the context of soliton lattice formation.

## 2. Methods

A single crystal of DyFeO_3_ was grown using the crucible-free zone melting method with optical heating in a mirror furnace FZ-4000 (Crystal System Co., Japan). The resulting 6 mm diameter and 17 mm length cylindrical crystal with the *b* axis aligned with the cylinder axis was cut into two half-cylinders, such that the large flat surface is the (001) crystal face.

Neutron diffraction measurements (Schmalzl *et al.*, 2024 ▸) were carried out on the IN12 cold-neutron three-axis spectrometer at the Institut Laue–Langevin (ILL), with a wavelength of 5.463 Å and a velocity selector upstream from the monochromator. A high-resolution diffraction setup was used, with a pyrolytic graphite (PG) (002) monochromator and a PG(002) analyser operating in optimal vertical focusing and no horizontal focusing mode. Horizontal collimation was achieved with 20′ collimators placed before and after the sample. The sample was mounted in an ILL Orange Cryostat with the *H*0*L* reflections in the scattering plane. The sample was oriented on the neutron Laue diffractometer OrientExpress at the ILL.

The high resolution provided by cold neutrons was required to separate the satellite reflections. However, the 1/*e* penetration depth of DyFeO_3_ for 5.463 Å neutrons is 0.18 mm, which results in collection of the signal effectively only from the crystal surface. The flat sample surface perpendicular to the *c* axis allows us to maximize the intensity of the 001 reflection by reducing the shadowing effect.

## 3. Results

**Figure 1:**
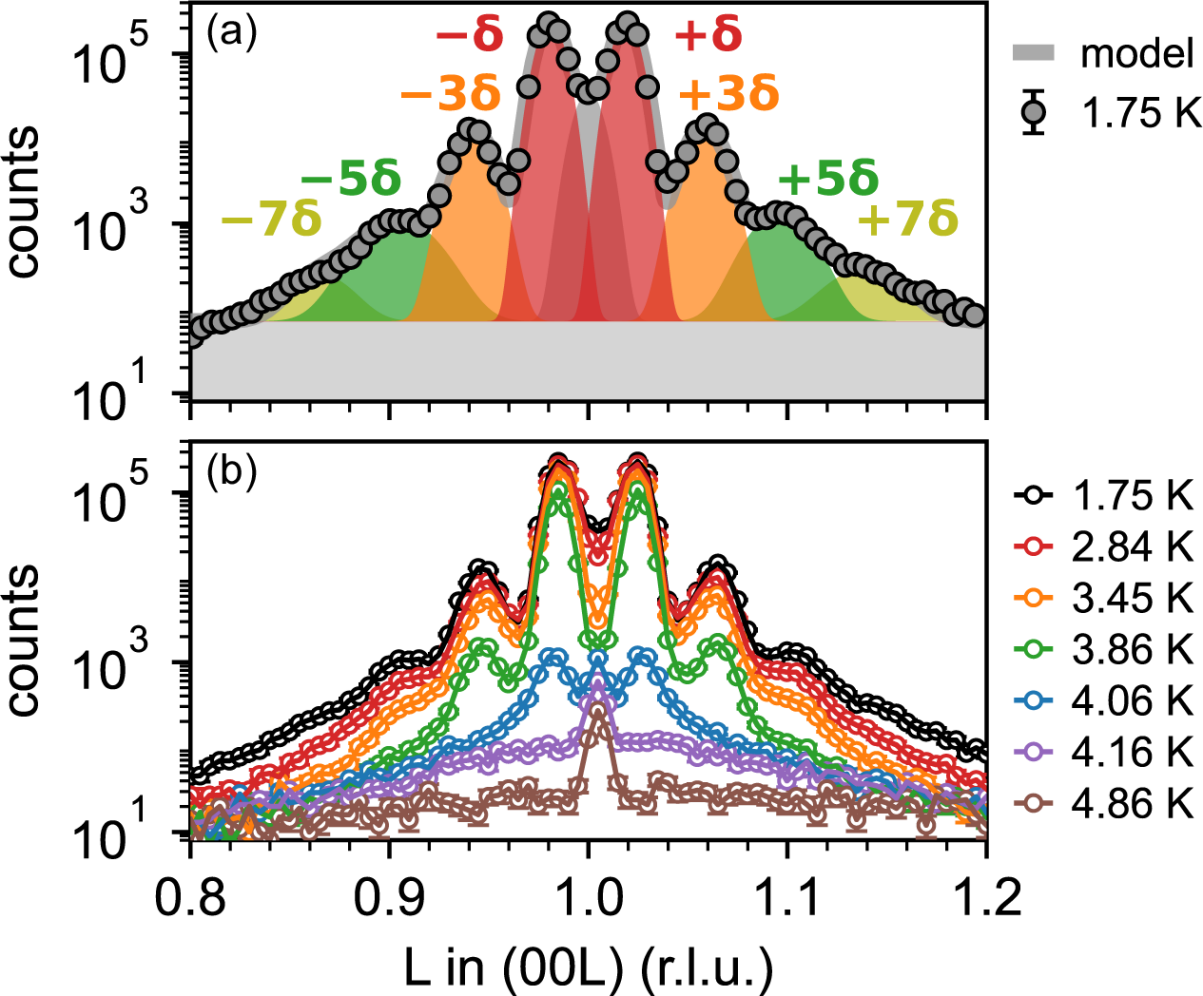
Neutron scattering intensities on the (00*L*) points in reciprocal space of single-crystal DyFeO_3_. They are shown on a logarithmic scale. (*a*) Representative scan at 1.75 K. Fits of odd magnetic satellite reflections up to seventh order are shown with coloured Gaussians, and the background is shown with a grey rectangle. (*b*) Selected scans in the temperature range 1.75–4.86 K.

A representative *L* scan across the 001 reflection at 1.75 K is shown in Fig. 1 ▸(*a*). It reveals a comb of satellite reflections indexed with the modulation vector (0, 0, δ) with δ = 0.0197 (1) reciprocal-lattice units (r.l.u.), revealing the odd harmonics of the underlying modulation. Satellites up to the seventh order are resolved, as well as the zero-order commensurate contribution. Selected scans in the temperature range 1.75–4.86 K are shown in Fig. 1 ▸(*b*). For each temperature, a set of nine Gaussians, representing the individual satellites together with a flat background, were fitted. Eight of these represent magnetic satellite contributions at ±δ, ±3δ, ±5δ and ±7δ with respect to the 001 reflection. The positions of the satellites were fitted with a single global δ parameter, multiplied by the order of the satellites. Left- and right-hand satellites of the same order were restricted to share the same intensities and FWHMs. The final Gaussian, centred at *L* = 1, represents the commensurate contribution.

**Figure 2:**
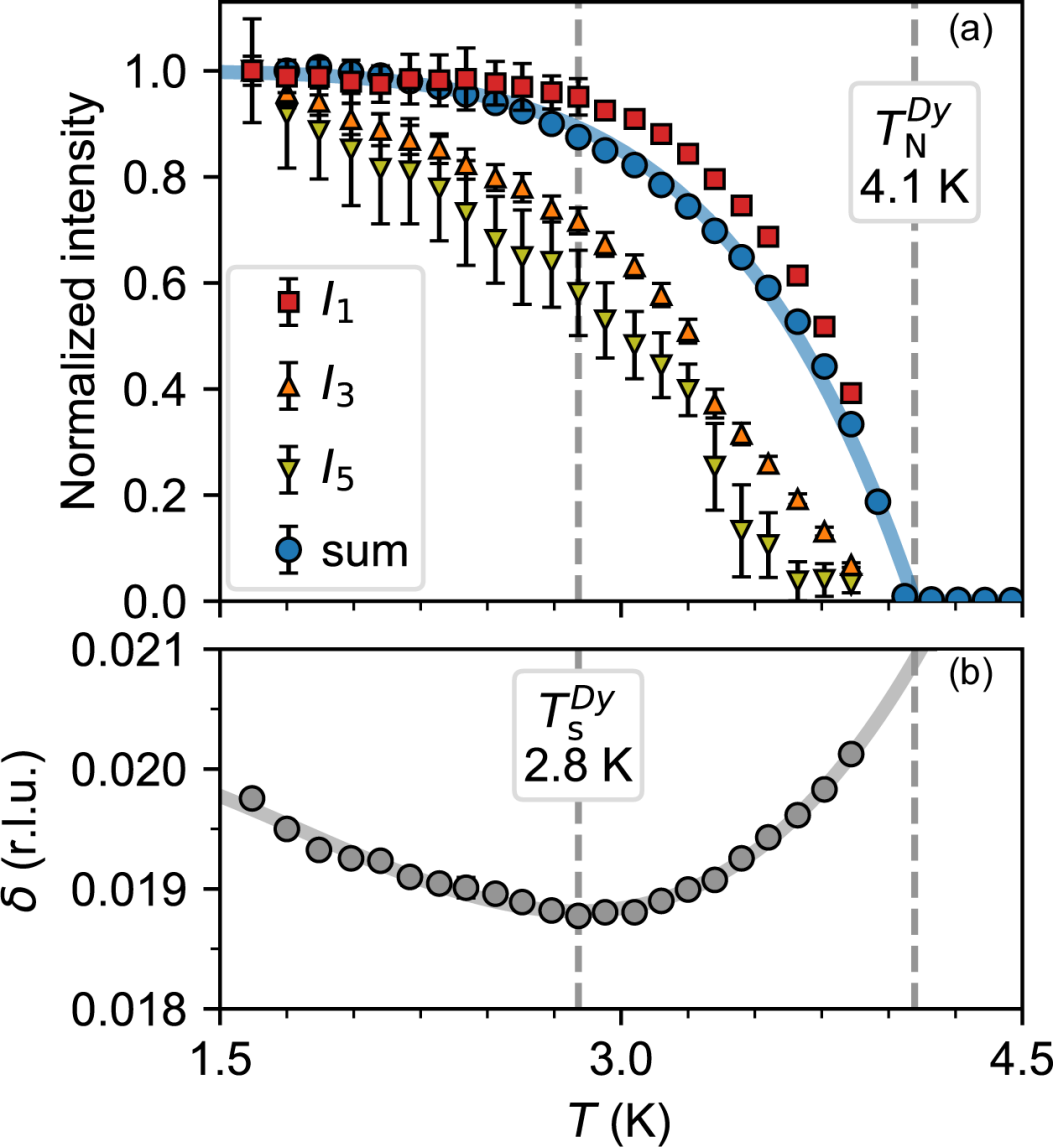
Temperature dependence of key parameters describing the DyFeO_3_ incommensurate magnetic ordering. (*a*) Normalized integrated intensities of the sum and first- (*I*_1_), third- (*I*_3_) and fifth-order (*I*_5_) satellite reflections. (*b*) Modulation vector (0, 0, δ) length. The saturation temperature of the first-order satellites, 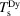 = 2.8 K, corresponds to the minimum of the modulation vector length δ = 0.0187 (1) r.l.u. Note that the intensities of the third- and fifth-order satellite reflections are not saturated down to the lowest temperatures measured.

The temperature dependence of the normalized (to the value of 1 at 1.62 K) integrated and first-, third- and fifth-order satellite reflection intensities is shown in Fig. 2 ▸(*a*). Fig. 2 ▸(*b*) presents the modulation vector length as a function of temperature. Below 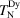
 = 4.1 K the intensities of all satellites are increasing. The higher the order of the satellite, the weaker the increase in intensity with decreasing temperature, as visual­ized in Fig. 2 ▸(*a*). The saturation of the intensity of the first-order satellite reflections occurs at the characteristic saturation temperature 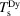
 ≃ 2.8 K. In contrast, the higher-order satellite reflections are not saturated down to a temperature of 1.62 K. Below 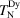, the modulation vector length decreases with decreasing temperature, reaching a minimum of 0.0187 (1) r.l.u. at 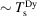, and then increases with further decreasing temperature as displayed in Fig. 2 ▸(*b*).

## 4. Discussion

**Figure 3:**
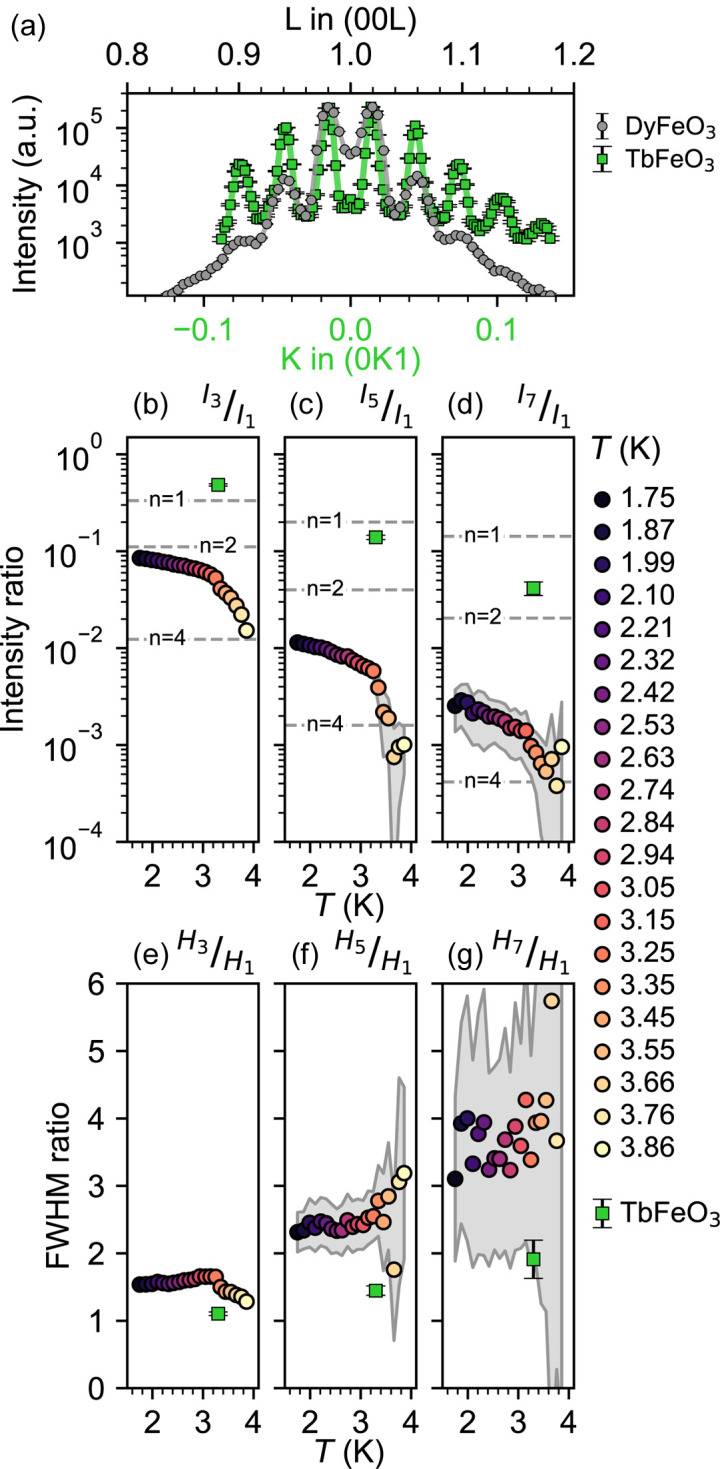
(*a*) Comparison of the (00*L*) scan of DyFeO_3_ at 1.75 K with the (0*K*1) scan of TbFeO_3_ at 3.3 K and 2 T magnetic field applied along the *c* axis from Fig. 2(*b*) of Artyukhin *et al.* (2012 ▸). (*b*), (*c*) and (*d*) Temperature dependence of intensities *I*_*p*_, and (*e*), (*f*) and (*g*) temperature dependence of FWHMs *H*_*p*_, of (*b*) and (*e*) third-, (*c*) and (*f*) fifth- and (*d*) and (*g*) seventh-order (*p*th) satellite reflections of DyFeO_3_ incommensurate ordering (circles), normalized to the corresponding values of the first-order satellite reflection. In panels (*b*)–(*g*), uncertainties are given as grey ribbons that extend to one standard deviation in either direction. Analogous values extracted from Fig. 2(*b*) of Artyukhin *et al.* (2012 ▸) are displayed as squares for comparison.

Incommensurate magnetic order in orthoferrites is uncommon, as apart from DyFeO_3_ an incommensurate ordering with high-order magnetic satellite reflections has only been reported for TbFeO_3_ (Artyukhin *et al.*, 2012 ▸). While the incommensurate order seems to be the ground state of DyFeO_3_, in TbFeO_3_ it appears as a pocket phase in the temperature range ∼2.7 < *T* < ∼3.5 K when a magnetic field stronger than 1 T is applied along the *c* axis. A comparison of DyFeO_3_ and TbFeO_3_ scans along the modulation vector directions is provided in Fig. 3 ▸(*a*). The incommensurate order in TbFeO_3_ is described as a soliton lattice, rooted in the magnetic ordering of the Tb sublattice. Within one unit cell, Tb ions order in an antiferromagnetic 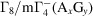 state, with A_*x*_ = 6.5 µ_B_ and G_*y*_ = 5 µ_B_ labelled with the order parameter η. On larger length scales the antiferromagnetic domains arise with a very regular length of ∼360 Å (67 unit-cell lengths) along the *b*-axis direction. Thus, the order parameter η changes between ±1 in alternating domains, with sharp jumps marking domain walls with a small extent in space. This domain structure results in satellite reflections with high-order harmonics, called the soliton lattice due to its regular size. As the magnetic order of the Dy sublattice in DyFeO_3_ is antiferromagnetic with moments lying within the *ab* plane (Ritter *et al.*, 2022 ▸; Biswas *et al.*, 2022 ▸; Mareschal & Sivardière, 1969 ▸), it is qualitatively similar to the case of Tb in TbFeO_3_.

Despite these similarities, there are significant differences between the rare earth magnetic orderings in DyFeO_3_ and TbFeO_3_. Incommensurate ordering seems to be the ground state for DyFeO_3_, but for TbFeO_3_ it requires an external magnetic field. The modulation vector of DyFeO_3_ is along the *c* axis, while that of TbFeO_3_ is along the *b* axis. In both DyFeO_3_ and TbFeO_3_, the rare earth magnetic moments lie in the *ab* plane, which means that, in DyFeO_3_, Dy^3+^ magnetic moments are perpendicular to the modulation vector, and in TbFeO_3_, Tb^3+^ magnetic moments have components both perpendicular and parallel to the modulation vector. Across the magnetic phase transition in TbFeO_3_, the length of the incommensurate part of the modulation vector length δ increases from zero to δ ≃ 0.015 r.l.u.; in DyFeO_3_, however, it only depends slightly on temperature, 0.0187 (1) ≤ δ < ∼0.02 r.l.u. [Fig. 2 ▸(*b*)]. For TbFeO_3_ (Artyukhin *et al.*, 2012 ▸), the intensities of higher-order satellites decrease very slowly with satellite order, while their FWHMs increase very slowly with satellite order, as shown in Fig. 3 ▸. This is different in DyFeO_3_, where much larger dependencies of satellite intensities [Figs. 3 ▸(*b*)–3 ▸(*d*)] and widths [Figs. 3 ▸(*e*)–3 ▸(*g*)] on satellite order were observed.

As noted in the *Results* section[Sec sec3], the saturation of the intensity of the first-order satellite reflections in DyFeO_3_ occurs at the characteristic temperature 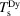 ≃ 2.8 K, which corresponds to the temperature of the δ(*T*) minimum marked with a dashed vertical line in Fig. 2 ▸. This satellite intensity measures the volume of the magnetically ordered Dy regions. The ordered regions grow in size from 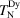 to 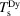, and 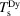 marks the temperature when magnetic Dy order spans the whole crystal. Note that within a Dy magnetically ordered region the soliton lattice exists with its signature regular domain structure. As the modulation length is inverse to the domain size, the domains within the soliton lattice grow, reaching a maximum length of ∼204 Å at the saturation temperature 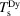. However, at 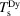 the Dy magnetic order is not settled. While the intensity of the first-order satellite is saturated between 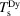 and the base temperature 1.62 K, the intensity of the third- and fifth-order satellites increases with decreasing temperature [Fig. 2 ▸(*a*)], suggesting subtle changes in the soliton lattice. According to the analysis of the intensity ratios of the magnetic satellites, we propose that in this temperature range this concerns the fine structure of the domain walls.

The intensities of the high-order satellites reduce with increasing temperature faster than those of the first satellite. In Figs. 3 ▸(*b*)–3 ▸(*d*) dashed lines mark the values expected for the relation *I*_*p*_/*I*_1_ = 1/*p*^*n*^ for *n* = 1, 2, 4. The value *n* = 2 corresponds to the square modulation of the order parameter, while larger values of *n* may describe a situation where the order parameter changes smoothly across the domain wall, such that the domain walls extend in length. The domain walls form a buffer layer between adjacent antiferromagnetic domains with a reduced magnetization of the Dy sublattice. Figs. 3 ▸(*b*)–3 ▸(*d*) show that the measured satellite intensities fall between the values *n* = 2 and *n* = 4 and become close to the square mod­ulation (*n* = 2) at low temperatures. With decreasing tem­perature, the relation tends towards lower *n* values, suggesting that the domain walls reduce in size. Further, the satellite intensities for TbFeO_3_ fall close to the *n* = 1 case. An extended analysis (Artyukhin *et al.*, 2012 ▸) shows that this calls for an extension of the simplistic model employed here but is qualitatively consistent with the square modulation of the order parameter.

A recent report on single-crystal neutron diffraction measurements of DyFeO_3_ (Nikitin *et al.*, 2025 ▸) shows a temperature evolution of the 001 magnetic satellite reflections similar to Fig. 2 ▸. The authors use a similar model to that of Artyukhin *et al.* (2012 ▸) and show that magnetic domains exhibit a log-normal distribution of size with a strongly reduced magnetization at the domain walls. According to Nikitin *et al.* (2025 ▸), the widths and intensities of satellite reflections correspond to the variance of the distribution of domain lengths and spatial extent of the domain walls, respectively. While Nikitin *et al.* (2025 ▸) applied their model to follow the evolution of the soliton lattice as a function of the applied field, we can utilize it to follow the evolution as a function of temperature. This shows that the domain length distribution does not change significantly with temperature, as evidenced by the satellite reflection width in Figs. 3 ▸(*e*)–3 ▸(*g*), while the spatial extent of the domain walls reduces strongly with decreasing temperature, as the satellite reflections increase with decreasing temperature [Figs. 3 ▸(*b*)–3 ▸(*d*)]. This is consistent with our findings, which are based solely on an analysis of reflection widths and intensities.

The DyFeO_3_ modulation vector length varies between different studies. Reports on single crystals give 0.0149–0.0169 r.l.u. (Nikitin *et al.*, 2025 ▸) and 0.0187–0.0205 r.l.u. [Fig. 2 ▸(*b*), this study], while reports on powder samples give 0.0173 (5) r.l.u. at 2 K (Biswas *et al.*, 2022 ▸) and 0.0265–0.0290 r.l.u. (Ritter *et al.*, 2022 ▸). The ranges in length correspond to the investigated temperature ranges. Note that the values reported by Ritter *et al.* (2022 ▸) are significantly (∼75%) greater than the correponding values in the work of Nikitin *et al.* (2025 ▸). Moreover, the reported 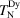 is inconsistent; it was reported for the range 3.7–4.7 K [Nikitin *et al.* (2025 ▸), Biswas *et al.* (2022 ▸) and Ritter *et al.* (2022 ▸), and citations therein]. As such, the magnetic properties of DyFeO_3_ seem to be sensitive to the sample form, preparation method, presence of impurities or temperature/field treatment protocol.

The Fe^3+^ magnetic order below 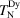 is still under debate. The same Fe^3+^ magnetic order 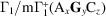 above and below 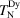 was reported by Wang *et al.* (2016 ▸) and Ritter *et al.* (2022 ▸), while Biswas *et al.* (2022 ▸) reported a 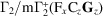 order below 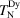. Additionally, on the basis of measurements of the thermal conductivity, magnetization and electric polarization, Zhao *et al.* (2014 ▸) reported the unresolved magnetic structure labeled ‘Fe_III_’, stabilized by an applied magnetic field below 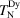, and suggested that it could be a ground state of the Fe^3+^ magnetic sublattice below ∼500 mK. Here, in the investigated temperature range of 1.62–4.86 K, the 001 commensurate reflection is present as shown in Fig. 1 ▸(*b*). Its intensity is relatively weak around and above 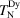 but it was not visible in a test measurement at a different wavelength, proving that it originates from multiple scattering and showing the high crystalline quality of the sample. Below the temperature of saturation of the first-order satellite reflections, 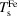 = 2.8 K, the commensurate reflection 001 is relatively strong. In the *Pbnm* space group, the 001 nuclear reflection is extinct. This commensurate peak could originate from the presence of a weak A_*x*_ component of Fe^3+^ magnetic ordering, allowed in the 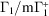 phase, or might be caused by the weak commensurate component of the Dy^3+^ ordering.

## 5. Conclusions

We report on the incommensurate magnetic ordering of DyFeO_3_ below 4.1 K using high-resolution neutron diffraction. We have investigated the characteristics of the magnetic satellites of the 001 reflection as a function of temperature. The results were compared with the soliton lattice model in TbFeO_3_. We have analysed the formation of the soliton lattice as a function of temperature, showing that first the magnetically ordered Dy regions grow in size to populate the whole volume of the crystal, while later the domain walls reduce in size down to the lowest temperature measured. The magnetic properties of DyFeO_3_ seem to be sensitive to the sample form, preparation method or presence of impurities. Understanding the reasons for these differences could lead to the possibility of precisely controlling the DyFeO_3_ magnetism.

## Supplementary Material

DyFeO3 single-crystal neutron diffraction data collected on the IN12 instrument at the Institut Laue-Langevin, France: https://doi.org/10.5291/ILL-DATA.INTER-599

## Data Availability

Raw data from our experiment are stored on the Institut Laue–Langevin server. They are available under the link https://dx.doi.org/10.5291/ILL-DATA.INTER-599.
